# Comparing the Age-Friendliness of Different Neighbourhoods Using District Surveys: An Example from Hong Kong

**DOI:** 10.1371/journal.pone.0131526

**Published:** 2015-07-01

**Authors:** Moses Wong, Pui Hing Chau, Francis Cheung, David R. Phillips, Jean Woo

**Affiliations:** 1 Department of Medicine & Therapeutics, The Chinese University of Hong Kong, Shatin, N.T., Hong Kong, China; 2 School of Nursing, The University of Hong Kong, Pokfulam, Hong Kong, China; 3 Department of Applied Psychology, Lingnan University, Tuen Mun, N.T., Hong Kong, China; 4 Department of Sociology and Social Policy, Lingnan University, Tuen Mun, N.T., Hong Kong, China; UNIVERSITY OF LAUSANNE, SWITZERLAND

## Abstract

**Background:**

To address the age-friendliness of living environment in cities, the World Health Organization (WHO) launched the “Age-friendly cities” (AFC) initiative in 2005. To date, however, no universal standard tool for assessing age-friendliness of a community has been agreed.

**Methodology:**

Two quantitative studies on AFC conducted in two Hong Kong districts—Sha Tin and Tuen Mun—were compared. A total of 801 residents aged ≥50 years were interviewed using structured questionnaires based on the WHO’s AFC criteria. District-wide differences in age-friendliness were compared on the basis of eight domain scores. Multiple linear regression was used to examine associations with demographic and socio-economic characteristics. The provision of services and amenities was also compared to help explain the difference in domain scores.

**Results:**

Variations in mean domain scores were observed in both districts. Sha Tin showed significantly lower scores in outdoor spaces and buildings, transportation, social participation, respect and social inclusion, civic participation and employment, communication and information, as compared with Tuen Mun. Although a significantly higher score on the housing domain was observed in Sha Tin, differences in community and health services domains were insignificant. Socio-demographic factors, such as age group, gender, area of residence, type of housing, experience of elderly care, employment status, self-rated health and income, were associated with domain scores. However, variations in services and amenities provision appeared not to be strongly associated with district-wide difference in domain scores.

**Conclusions:**

District differences in public opinions towards age-friendly characteristics were observed in this study. Except for two of the eight domains, Sha Tin had significantly lower scores than Tuen Mun. Some socio-demographic indicators seemed predictive to the differences. Paradoxically, Sha Tin had better services and infrastructure and higher socio-economic status, but lower age-friendliness. This warrants detailed research on psychosocial factors that may influence residents’ perceptions of local environments.

## Introduction

Populations have been ageing rapidly worldwide and especially in the richer countries of the Asia-Pacific [1]. The fast growing number of older people living in cities and communities warrants attention from the multiple perspectives of medical and health services and social and public policy fronts [2–4]. Urbanization and planning strategies now often encourage and sometimes require local governments and civic organizations to develop built and social environments able to accommodate the changing aspirations of all residents and responsive to the changing needs of urban living [5, 6]. At the forefront of trends such as ageing in place, which requires communities to be accessible to their inhabitants, is the growing interest in cities’ ‘age-friendliness’. The age-friendly city concept was based partly on the active ageing framework, defined by the World Health Organization (WHO) as “the process of optimizing opportunities for health, participation, and security in order to enhance quality of life as people age” [7]. It is increasingly believed that a supportive environment would be beneficial to health and well-being as it enables residents to grow older actively and successfully in place, without the need to move [8].

To address the age-friendliness of living environment in cities, the WHO launched the “Age-friendly cities” initiative in 2005. The formal programme started in 2006 with 33 cities from 22 countries worldwide participating in a focus group research project, where older persons expressing their opinions about age-friendly features. Eight domains summarizing age-friendly city features were identified: outdoor spaces and buildings; transportation; housing; social participation; respect and social inclusion; civic participation and employment; communication and information; and community support and health services [9]. The publication *Global Age-friendly Cities*: *a Guide* and a checklist of essential features of age-friendly cities were subsequently developed in 2007 for cities to use as a tool to assess their age-friendly city features and for future improvements [9, 10]. This guide also formed one of the principles for cities and communities to join the WHO Global Network of Age-Friendly Cities and Communities (GNAFCC), which was established to encourage the exchange of experience and learning among the members.

Gathering evidence for evaluation is one of the crucial steps towards policy formulation, bridging the gap between pilot and rollout phases of programmes, or moving from the stage of inspiration to those of strategy formulation and implementation. In the four-step network cycle of GNAFCC, “development of a baseline assessment of the age-friendliness of the city/community” is one of the key elements [11]. However, there has to date been no specific guidance developed by the WHO on the use of a standardized assessment tool, though the WHO Centre for Health Development (the Kobe Centre) is in the process of developing a new approach for cities to measure their age-friendliness based on a set of core indicators. For the purpose of data sustainability, the selected core indicators are mostly those available from routine data and existing statistical databases which would normally be obtained at the city/community level.

Whilst this approach will certainly enable monitoring and ‘macro-scale’ comparisons of cities or urban areas over time, specific surveys could be conducted to assess individuals’ opinions towards the age-friendliness of their community. While qualitative research methods are useful to identify local needs and area of improvement, quantitative research methods are also very useful to assess the level of age-friendliness of cities and trends over time, to evaluate programmes or programme elements that promote age-friendliness, and to enable comparisons with other communities. International experiences have identified key elements of age-friendly communities in various countries. The pioneer attempt was the Age-friendly New York City initiative in 2007, in which age-friendly characteristics were assessed and suggestions made for improvement. These included local factors such as ensuring pedestrian safety in neighbourhoods, improving affordability of housing and encouraging courtesy towards older persons, and offering opportunities for social interactions to avoid isolation, etc [12]. Other global initiatives also suggested the development of good and affordable public transport and housing provision, safe physical environments with barrier-free features, service proximity and social inclusiveness constitute important characteristics everywhere [13–16]. However, these findings are predominantly based on projects from North American and European countries. They are likely to have limited generalizability to high-rise, high density cities in the Asia-Pacific region. Here, research is scarce and the lack of a standardized assessment tool hinders cross-national and even intra-country comparisons.

Hong Kong has been relatively early in promoting the concept of age-friendly cities, starting in 2008 with a wide support from NGOs, governmental bodies, district councils, academic, professional associations, and businesses. A local report summarized some of the findings from the qualitative and quantitative research studies on assessing the age-friendliness in Hong Kong [17]. In 2010, the Hong Kong Council of Social Services (HKCSS) conducted eight focus groups to assess level of age-friendliness in Hong Kong corresponding to the eight domains. While a qualitative approach was useful in identifying good practices and areas for improvements, it does not quantify age-friendliness nor the relative importance of specific components or aspects. In 2013, the HKCSS proposed a set of 24 indicators (with 3 indicators in each domain) based on routine or official statistics. The concept was similar to the WHO’s approach of using core indicators, which are mainly collected at the community level, and small area comparisons were generally not supported. Using a questionnaire approach, groups of Age-Friendly Hong Kong ambassadors in various districts conducted surveys to assess perceived levels of age-friendliness on specific aspects or domains, and made recommendations. However, few of them covered all eight domains to enable comprehensive cross-district comparisons.

Attempts have therefore been made by teams from local universities to assess the age-friendliness of different districts in Hong Kong using a questionnaire approach that included questions covering all the eight domains. The questionnaire was developed-based on the WHO’s list of desirable features gathered from focus groups all over the world. The respondents were asked to answer the questions based on their personal experience and subjective perception. Since subjective perception may be influenced by other factors such as socioeconomic status and/or education, where possible we also collected objective data such as availability of health care, transport, employment etc. in order to examine whether domains in the WHO guidelines may be affected by individual factors. This constitutes the first attempt to examine the WHO guidelines in this manner. In 2011, the first environmental scan was carried out in a local district (Sha Tin) by the Department of Medicine and Therapeutics of The Chinese University of Hong Kong, and the Faculty of Social Sciences of The University of Hong Kong, with the support of CADENZA project [18]. Drawing on the experience of this first environmental scan, the Department of Sociology and Social Policy of The Lingnan University carried out similar study to investigate age-friendliness in another local district (Tuen Mun) in 2012 [19]. Sha Tin district and Tuen Mun district share some common characteristics. First, both districts are located in the New Territories, previously considered as rural areas in the old days but have been developed as busy new towns of around half a million persons. In the 1970s, Sha Tin New Town was developed and Tuen Mun New Town was developed a little later, both sharing similar planning characteristics, by which they were constructed around a town centre. Subsequently, a relatively smaller Ma On Shan new town was developed within Sha Tin district, including larger private housing estates but also some commercial zones and hospital facilities. In Tuen Mun, a similar outlier new area was developed, the Hong Kong Gold Coast, which has also grown since the early 1980s, with private housing estates, some public housing, and tourist attractions including a hotel, beach, shopping mall and a marina. In terms of demographic characteristics, Sha Tin district has a slightly larger population (643,000) than Tuen Mun district (486,300), each with respectively 12.6% and 10.6% of population aged 65 and above [20]. Education and income levels tended to be higher in Sha Tin (percentage of district population with secondary education and above was 81.1%; median household income, HK$24,900, US$1 = HK$7.75) than those in Tuen Mun (77.2%, and HK$20,000 respectively), partly because of the higher concentration of public housing estates in Tuen Mun. These two districts have interesting similarities and differences in terms of their geographic and demographic characteristics, and serve as good comparators. This paper aimed to examine the differences in age-friendliness by comparing the findings from these two questionnaire studies. We also examined whether any differences were associated with demography and socio-economic characteristics as well as objective measurements of services and facilities in respective districts.

## Materials and Methods

### Data collection

This study was based on the analysis of two surveys conducted in Sha Tin and Tuen Mun, which adopted a quantitative approach to collect primary data on residents’ opinions towards the age-friendliness characteristics. A structured questionnaire was developed by the Sha Tin team based on a local adaptation of the WHO age-friendly city guidelines [9, 10], and a shortened and modified version was adopted in the Tuen Mun data collection to enable direct comparisons. In the Sha Tin study, 85 aspects under the 8 domains were incorporated into the questionnaire, whereas 50 aspects under the 8 domains were covered in Tuen Mun data. Respondents rated their feelings towards the AFC items on a 6-point Likert scale, ranging from 1 (‘strongly disagree’) to 6 (‘strongly agree’), with higher scores indicating greater age-friendliness.

The two studies recruited convenience samples of residents aged ≥35 years living in Sha Tin and aged ≥50 years in Tuen Mun. Data were collected by face-to-face questionnaire surveys and some self-administered questionnaire from February to September 2011 in Sha Tin, and from June to August 2012 in Tuen Mun. Trained research assistants conducted face-to-face interviews, while some more literate subjects responded to self-administered questionnaires with assistance from the trained research assistants. Interviews were conducted in parks, housing estates, and public areas in Sha Tin; while parks, markets and public recreational areas were the major sites where Tuen Mun participants were interviewed. Geographical variations were considered so that participants were drawn from various environments in both districts. Respondents’ demographic and socio-economic characteristics were collected. Both studies over-sampled older people and residents living in town centres and non-town centres to facilitate sub-group analyses. Information on objective measurements in terms of services and facilities were gathered from multiple source of official statistics.

### Data analysis

The two district scans incorporated 48 directly comparable aspects which were used in the cross-district comparison. Domain scores were estimated by the average of the scores of the individual components under the corresponding domains. Domain scores were calculated only if over half of the aspects under that domain had valid responses.

Only those aged ≥50 years from both sites were included for the analysis. The inclusion of the age group 50–64 allowed the examination of the views of “soon-to-be old” group, in addition to those of existing older age groups. The samples were grouped into three age groups for analysis (50–64, 65–79, and ≥80). Areas of residences of the respondents were grouped into two sub-areas (town centre, and non-town centre). This incorporated the town centre in Sha Tin and the inner city in Tuen Mun, where services and facilities were concentrated and the flow of people was larger. Non-town centre referred to the new town areas of Ma On Shan in Sha Tin and the middle class residential area of the Gold Coast in Tuen Mun.

Chi-square test was used to examine differences in sample characteristics. Since the sample composition in the two studies differed somewhat, when examining the difference of AFC domain scores between the two districts, multiple linear regression was used to adjust for demographic and socio-economic characteristics that showed significant differences.

To identify socio-demographic predictors of each of the AFC domain scores, multiple linear regression was applied to the combined data from Sha Tin and Tuen Mun. The factors included age group, gender, area of residence (town centre, non-town centre), type of housing (private, public), education level (below primary, primary, secondary, tertiary), marital status (currently married, others), employment status (retired, employed, economically inactive except retired), self-rated health (poor, fair, good, very good/excellent), experience of taking care of older persons, self-perceived disposable income (insufficient, enough, sufficient), and household income (<HK$15,000, ≥HK$15,000). A backward elimination procedure was used to remove insignificant factors until all remaining variables became significant in the final model. SPSS version 20 was used in all statistical procedures. A significant level of 5% was adopted.

To explore whether AFC domain scores varied by objective measurements or personal perception, a non-exhaustive list was developed regarding facilities and services available in the two districts. The measurements included population profiles, availability of open spaces and greenery, numbers of shopping malls and markets, transport networks and types of transportations, number of housing units, number of various public facilities allowing social participation, number of elderly abuse cases reflecting respect and social inclusion, as well as community and health services. Multiple data sources were used, including those from the Census and Statistics Department, the Social Welfare Department, District Councils and the Planning Department. Since Sha Tin has a slightly larger population than Tuen Mun, the statistics were adjusted to as per 1,000 population aged ≥65y.

### Ethics Statement

Ethics approvals were obtained from The Chinese University of Hong Kong and The Lingnan University. Written informed consent and verbal informed consent were sought respectively in Sha Tin and Tuen Mun from the respondents.

## Results

A total of 311 and 490 completed questionnaires from Sha Tin and Tuen Mun respectively were included in this comparison study. Of the respondents in Sha Tin study, 61.4% were aged 65 years or above and 60.3% were male; while respective figures were 64.7% and 45.0% in Tuen Mun (Table 1). In the Sha Tin sample, 60.8% of the respondents were town centre residents and 79.0% of Tuen Mun respondents lived in town centre. Chi-square test results showed that the socio-demographic characteristics were significantly different between the two samples, except for age group (p = 0.175), type of housing (p = 0.325), and disposable income (p = 0.858) (Table 1).

**Table 1:** Socio-demographic characteristics of the respondents in Sha Tin and Tuen Mun.

	Sha Tin	Tuen Mun	P-value*
Number of respondents	311	490	
	N (%)	N (%)	
***Age group***			0.175
50–64	120 (38.6%)	173 (35.3%)	
65–79	134 (43.1%)	243 (49.6%)	
≥80	57 (18.3%)	74 (15.1%)	
***Gender***			<0.001
Male	187 (60.3%)	220 (45.0%)	
Female	123 (39.7%)	269 (55.0%)	
***Area of residence***			<0.001
Town centre	189 (60.8%)	387 (79.0%)	
Non-town centre	122 (39.2%)	103 (21.0%)	
***Type of housing***			0.325
Private	84 (27.9%)	121 (24.7%)	
Public	217 (72.1%)	368 (75.3%)	
***Education level***			<0.001
Below primary	34 (11.5%)	108 (22.2%)	
Primary	126 (42.7%)	195 (40.0%)	
Secondary	103 (34.9%)	160 (32.9%)	
Tertiary	32 (10.8%)	24 (4.9%)	
***Marital status***			0.013
Currently married	239 (80.5%)	355 (72.6%)	
Others	58 (19.5%)	134 (27.4%)	
***Employment status***			0.001
Retired	202 (67.8%)	275 (56.7%)	
Active population	47 (15.8%)	72 (14.8%)	
Inactive (except retired)	49 (16.4%)	138 (28.5%)	
***Self-rated health***			<0.001
Poor	38 (13.0%)	38 (7.8%)	
Fair	157 (53.8%)	192 (39.3%)	
Good	68 (23.3%)	189 (38.7%)	
Very good/Excellent	29 (9.9%)	70 (14.3%)	
***Elderly care experience***			<0.001
No	160 (54.8%)	329 (67.4%)	
Yes	132 (45.2%)	159 (32.6%)	
***Disposable income***			0.858
(Very) insufficient	52 (17.8%)	87 (17.8%)	
Enough	189 (64.7%)	309 (63.2%)	
(Very) sufficient	51 (17.5%)	93 (19.0%)	
***Household income***			0.003
< HKD 15,000	196 (69.3%)	386 (78.9%)	
≥ HKD 15,000	87 (30.7%)	103 (21.1%)	

*P-value from Chi-square test.

The mean domain scores varied among the 8 domains in both studies (Table 2). Regardless of adjustment for differences in sample characteristics, significantly lower domain scores (p<0.001) were observed in Sha Tin as compared with Tuen Mun in six of the eight domains: outdoor spaces and buildings (4.17 in Sha Tin vs 4.40 in Tuen Mun); transportation (4.20 vs 4.36); social participation (4.09 vs 4.54); respect and social inclusion (3.56 vs 4.11); civic participation and employment (3.00 vs 3.77); communication and information (3.74 vs 4.38). By contrast, a significantly higher domain score was observed in Sha Tin as compared with Tuen Mun in the housing domain (4.07 vs 3.77, p<0.001). There was no statistically significant difference in the community and health services domain (3.34 vs 3.49, p = 0.344). The transportation domain and the outdoor space and buildings domain ranked the highest in the Sha Tin survey, whilst the social participation domain and the outdoor space and buildings domain ranked the highest in Tuen Mun. The civic participation and employment and the community and health services domains ranked lowest in both studies. The housing domain was ranked as low as the civic participation and employment domain in the Tuen Mun study.

**Table 2:** Mean scores of Age-friendly City domains in Sha Tin and Tuen Mun.

	Sha Tin		Tuen Mun		Difference (Sha Tin–Tuen Mun)		P-value#
Domain	Mean	SD	Mean	SD	Raw	Adjusted#	
Outdoor spaces and buildings	4.17	0.69	4.40	0.59	-0.24	-0.22	<0.001
Transportation	4.20	0.62	4.36	0.49	-0.16	-0.16	<0.001
Housing	4.07	0.88	3.77	0.76	0.31	0.35	<0.001
Social Participation	4.09	0.88	4.54	0.65	-0.45	-0.43	<0.001
Respect and social inclusion	3.56	0.85	4.11	0.72	-0.55	-0.51	<0.001
Civic participation and employment	3.00	1.04	3.77	0.81	-0.78	-0.77	<0.001
Communication and information	3.74	0.83	4.38	0.65	-0.65	-0.61	<0.001
Community and health services	3.34	0.92	3.49	0.77	-0.14	-0.06	0.344

# P-value and the district difference were based on multiple linear regression, adjusting for gender, area of residence, marital status, education level, experience of taking care of older persons, employment status, self-rated health, and household income.

Table 3 shows the predictors of age-friendliness domain scores retained by backward elimination procedure in multiple linear regressions (Table 3). For outdoor spaces and buildings domain, people who rated this domain low tended to be male, aged 50–64, living in public housing, and with poor or fair self-rated health. In terms of transportation, people who rated this domain low were aged 50–64, and had insufficient or just enough disposable income. On housing, people aged 50–64, who lived in private housing, and who had insufficient disposable income rated this domain low. For social participation, people who rated this domain low lived in non-town centre, and had poor or fair self-rated health. For respect and social inclusion, people who were male, and who had monthly household income ≥HK$15,000, rated this domain low. For civic participation and employment, people living in non-town centre, who were retired, and had monthly household income ≥HK$15,000 rated this domain low. For communication and information, people who rated this domain low were male, aged 80 and above, lived in non-town centre, had elderly care experience, and had poor or fair self-rated health. For community and health services, people who rated this domain low lived in non-town centre.

**Table 3:** Multiple linear regressions with backward elimination procedure assessing significant factors associated with domain scores of age-friendliness.

	Outdoor spaces and buildings		Transportation		Housing		Social participation		Respect and social inclusion		Civic participation and employment		Communication and information		Community and health services	
	Coef	95% CI	Coef	95% CI	Coef	95% CI	Coef	95% CI	Coef	95% CI	Coef	95% CI	Coef	95% CI	Coef	95% CI
***Age group***																
50–64	-0.231	(-0.366,-0.097)	-0.157	(-0.271,-0.044)	-0.326	(-0.497,-0.155)	-0.087^§^	(-0.254,0.079)	—		—		0.049	(-0.117,0.216)	-0.156^§^	(-0.332,0.021)
65–79	0.007	(-0.121,0.135)	0.018	(-0.091,0.127)	-0.048	(-0.211,0.116)	0.121^§^	(-0.039,0.281)	—		—		0.173	(0.015,0.331)	0.061^§^	(-0.109,0.231)
≥80	0		0		0		0						0		0	
***Gender***																
Male	-0.122	(-0.211,-0.033)	—		—		—		-0.184	(-0.297,-0.070)	—		-0.176	(-0.286,-0.066)	—	
Female	0								0				0			
***Area of residence***																
Town centre	—		—		—		0.126	(0.002,0.249)	—		0.191	(0.036,0.347)	0.148	(0.025,0.270)	0.195	(0.064,0.326)
Non-town centre							0				0		0		0	
***Type of housing***																
Private	0.119	(0.017,0.222)	—		-0.158	(-0.293,-0.023)	—		—		—		—		—	
Public	0				0											
***Education level***																
Below primary	—		—		—		—		—		—		—		—	
Primary	—		—		—		—		—		—		—		—	
Secondary	—		—		—		—		—		—		—		—	
Tertiary																
***Marital status***																
Currently married	—		—		—		—		—		—		—		—	
Others																
***Employment status***																
Retired	—		—		—		—		—		-0.302	(-0.470,-0.134)	—		—	
Active population	—		—		—		—		—		-0.159	(-0.391,0.073)	—		—	
Inactive (except retired)											0					
***Self-rated health***																
Poor	-0.265	(-0.456,-0.073)	—		—		-0.247	(-0.490,-0.004)	—		—		-0.402	(-0.636,-0.167)	—	
Fair	-0.164	(-0.306,-0.022)	—		—		-0.294	(-0.473,-0.116)	—		—		-0.247	(-0.421,-0.073)	—	
Good	-0.019	(-0.165,0.127)	—		—		-0.112	(-0.295,0.070)	—		—		-0.13	(-0.309,0.049)	—	
Very good/Excellent	0						0						0			
***Elderly care experience***																
No	—		—		—		—		—		—		0.269	(0.155,0.382)	—	
Yes													0			
***Disposable income***																
(Very) insufficient	—		-0.268	(-0.394,-0.143)	-0.407	(-0.601,-0.212)	—		—		—		—		—	
Enough	—		-0.125	(-0.225,-0.025)	-0.03	(-0.183,0.123)	—		—		—		—		—	
(Very) sufficient			0		0											
***Household income***																
<15,000 HKD	—		—		—		—		0.143	(0.011,0.275)	0.182	(0.014,0.350)	—		—	
≥15,000 HKD									0		0					

Abbreviation: Coef = Coefficient

—Predictors removed in backward elimination procedure and not included in the final model.

^§^No significant subgroup difference despite age group was significant predictor in the final model (Overall p = 0.004).

Table 4 shows the provision of services and amenities of the two local environments. Sha Tin had more open space and greenery space, as well as major transportation infrastructure than Tuen Mun. By contrast, Tuen Mun had more public housing units (under different housing schemes) and more publicly funded hospital beds than Sha Tin. The numbers of amenities for social participations were similar in the two districts, although Tuen Mun had more parks though the size of the park was not reflected. The elder abuse rate was higher in Tuen Mun (0.75 per 1,000 older persons aged 65y and above) than that in Sha Tin (0.52 per 1,000 older persons aged 65y and above). With a broad comparison, Sha Tin appeared to show better indicators in terms of provision of services and infrastructure. However, these advantages did not necessarily lead to better satisfaction as reflected in the lower scores in most AFC domains in Sha Tin. It appeared that the sufficiency in services and infrastructure might not be associated with the differences in the AFC domain scores.

**Table 4:** Comparison of infrastructure, social amenities and services in Sha Tin and Tuen Mun.

	Sha Tin	Tuen Mun	Sha Tin	Tuen Mun
Domain				
Population (as of 2013)	643,000	486,300		
Population aged 65 and above	80,700	51,700		
Percentage of elderly aged 65 and above in district	12.6%	10.6%		
	**Total**		**Per 1,000 older persons aged 65y and above**	
**Outdoor spaces and buildings**				
Open space (area in hectare)	293.3	111.6	3.63	2.16
Green Belt (area in hectare)	1,389.5	743.5	17.22	14.38
Conservation area (area in hectare)	11.9	NA	0.15	NA
Site of scientific interest (area in hectare)	2.5	42.7	0.03	0.83
Country park (area in hectare)	0.6	NA	0.01	NA
Number of major shopping malls	17	31	0.21	0.60
**Transportation**				
Major road (area in hectare)	315.7	178.3	3.91	3.45
Number of major trunk routes and traffic arteries	13	4	0.16	0.08
Number of tunnels	6	0	0.07	0.00
Number of stations of rail service	13	44	0.16	0.85
Number of bus routes	121	63	1.50	1.22
Number of minibus routes	42	20	0.52	0.39
Number of ferry piers	0	1	0.00	0.02
Number of water transport routes	NA	4	NA	0.08
**Housing**				
Number of public estates (including Tenant Purchase Scheme)	21	13	0.26	0.25
Number of public rental units (including Tenant Purchase Scheme)	65,337	58,890	809.63	1139.07
Number of residents in public housing (including Tenant Purchase Scheme)	171,100	150,700	2120.20	2914.89
Number of Home Ownership courts	25	18	0.31	0.35
Number of Home Ownership units	50,119	40,116	621.05	775.94
Number of private estates	56	44	0.69	0.85
**Social participation**				
Number of parks	5	28	0.06	0.54
Number of recreational grounds	16	11	0.20	0.21
Number of sports complex	5	4	0.06	0.08
Number of swimming pool	3	3	0.04	0.06
Number of beach	0	7	0.00	0.14
Number of library	3	3	0.04	0.06
Number of community hall and plaza	12	10	0.15	0.19
Number of museum and historic site	11	14	0.14	0.27
Number of welfare service units managed or funded by SWD	49	34	0.61	0.66
**Respect and social Inclusion**				
Number of elderly abuse cases	42	39	0.52	0.75
**Community and health services**				
Number of General Out-patient Clinics	4	4	0.05	0.08
Number of hospitals and institutions run by Hospital Authority (HA)	4	3	0.05	0.06
Number of private hospital	1	0	0.01	0.00
Number of HA hospital beds	2,297	3,490	28.46	67.50
Number of private hospital beds	418	0	5.18	0.00
Magistracy	1	1	0.01	0.02
Government office	1	2	0.01	0.04
Police station	4	3	0.05	0.06
Fire station & Ambulance depot	7	7	0.09	0.14
Post office	11	6	0.14	0.12

Remarks: NA = statistics not available

## Discussion

This is the first attempt to assess quantitatively the age-friendliness of districts in Hong Kong where opinion had been sought from members of the community drawn from a wide range of ages from 50 years and older. The results showed some important district differences, even though Hong Kong is a small geographic area, with comprehensive planning authorities using similar standards. More interestingly, there are factors that are not solely dependent on WHO domains that appear to influence ratings.

With the exception of the housing domain and the community and health services domain, AFC scores were lower in Sha Tin compared with Tuen Mun. This finding was surprising since the Tune Mun population had lower income, lower education level, with a higher prevalence of people who were not actively employed. At the same time their self-rated health was higher compared with people in Sha Tin. Even though we adjusted for the differences in the socio-demographic characteristics of the two samples, the differences in domain scores persisted. When we examined whether this difference was attributable to differences in provision of services and amenities of the local environments, we found that, paradoxically, Sha Tin appeared to have better community provision in many objective aspects compared with Tuen Mun, despite the fact that town planning/service provision was undertaken according to the Hong Kong Planning Standards and Guidelines of the Planning Department in Hong Kong [21]. Sha Tin district appeared to have somewhat better outdoor physical environments in terms of open spaces and greenery, better road density and road traffic options. However, Sha Tin had considerably fewer public sites, such as parks, beaches and museums. The publicly funded housing and health services in Sha Tin were at a lower level than in Tuen Mun, in that relatively fewer public housing units and fewer public hospital beds were provided in Sha Tin. Nevertheless, the figures regarding provision of such district level infrastructure may not reflect actual utilization. For example we have not collected data regarding utilization of shopping malls and parks or other amenities by residents from neighbouring districts, or indeed from mainland China, in particular since both districts are within close proximity to the border with mainland China. Similarly cross district utilization of health care facilities may occur, although a previous study showed that this is only 20% or less [22].

Other factors that may contribute to the district differences may cover psychosocial factors that have not yet been taken into account in this survey. Indeed a separate analysis of the Tuen Mun data suggests that while most of the WHO domains were associated with psychological wellbeing, the strength of the association was not high, and it was suggested that other factors such as physical health may be more important determinants [19]. This was consistent with our findings which showed that Tuen Mun had higher scores in the respect and social inclusion domain than Sha Tin, yet Tuen Mun had a higher elder abuse rate than Sha Tin. Also, while Tuen Mun had more provision of public housing units, its housing domain score was significantly lower than that in Sha Tin. Our examination of the predictors of lower domain scores of AFC showed that lower scores were not always associated with the so-called deprived group. For example, those living in private housing rather surprisingly rated the housing domain worse than those respondents living in public housing; those who had higher monthly household incomes rated the respect and social inclusion domain and the civic participation and employment domain worse than those having lower incomes; those aged 50–64 rated the outdoor spaces and buildings domain, the transportation domain, and the housing domain worse than those aged 80 years or above. This study is suggesting considerable influences of socio-economic characteristics on the evaluation of AFC characteristics that warrants future research.

In view of the standardization imposed especially in new-build areas by the Hong Kong Planning Standards and Guidelines (Hong Kong Planning Department), it was expected that there should be relatively few notable differences in the age-friendliness of different residential areas (town centre vs non-town centre) in those domains related to infrastructure. Indeed, in line with expectations, area of residence was not an important predictor of scores in the three domains related to infrastructure and services provision (outdoor spaces and buildings, transportation, and housing). However, those living in non-town centres rated the remaining socially-orientated domains, such as social participation; and community and health services, worse. This was consistent with the statistics indicating that the provision of health care services in Sha Tin was rather less than in Tuen Mun.

A supplementary focus group study involving older people and their health service providers examining the ‘age-friendliness’ of primary care showed deficiencies in many domains according to the WHO age-friendly primary care criteria [22]. Future efforts towards improving ‘age-friendliness' may therefore need to identify and target vulnerable groups in each district, possibly using indicators such as the social vulnerability index [23]. The current study also suggests the wisdom of studying neighbourhoods (or subareas) instead of whole districts, since there are likely to be wide variations within each district. For age-friendliness, within each neighbourhood, environmental, social and physical aspects influence walkability, mobility, diet, social cohesion and depression [24]. It has also been recognized that the services and nature of local neighbourhoods tend to be more important for older groups than for most others, as older persons tend to have more limited activity spaces [25].

In terms of age-friendly characteristics, it could be concluded that Hong Kong already has many age-friendly features especially at a superficial level, in terms of walkability, accessibility of various services, community based social centres and services, and affordable public healthcare. Yet the surveys suggest that there is considerable room for improvement. Compared with the rapid developments of the age-friendly movement in many countries to date, summarized for example in a special issue of the Journal of Aging and Social Policy [5, 26, 27], Hong Kong still has to systematically embark on the four steps of establishing a mechanism to involve all older people (rather than just those affiliated with non-government run community centres), politicians in local government, government officials, service providers, industry and academia (for assessment and evaluation); a baseline assessment for all districts; developing a 3-year district wide action plan based on district assessment, and identifying indicators to monitor progress [11]. The conduct and analysis of these two environmental surveys of age-friendliness could serve as the first step.

Although the uptake of the AFC concept has been widespread and enthusiastic, several initiatives described in the Special issue of the Journal point to the challenges involved in sustaining this movement, such as low priority for funding, and older people being relegated to the fringe of society. Hong Kong faces similar challenges, such that for the movement to progress rather than fade away, the whole population needs to be aware of and engaged in ageing issues and resources included in all sectors covered by the WHO domains. For example, the setting up of a local association for retired persons (similar perhaps to USA’s AARP, the American Association of Retired Persons) could provide a cohesive ‘voice’; greater collaboration with industry and charitable foundations may also be helpful. The goal of age friendliness is worth pursuing because of the impact of these many environmental factors on self-rated physical health, depressive symptoms, maintaining physical activity and facilitating the desire to remain in one’s current home (to age in place). Older people in Hong Kong tend to have low civic participation and employment, as shown from the relatively low domain score in the surveys. A coordinated association for retired persons might help to enhance older persons’ morale and self-image and hence improve this AFC domain.

There are some limitations associated with the current study. First, only 48 out of the 85 aspects in the WHO AFC checklist could be directly compared between the two studies. The two pilot studies were based on convenience sample, so there might be selection bias. Importantly, older persons who were unable to access the open areas were excluded and the house-bound could be very much affected by AFC factors. The sampling strategy may introduce a bias towards omitting older people who may be frail and not likely to venture into public parks. The direction of the bias may be towards more favorable views as these participants may represent the more active group who are likely to face fewer societal obstacles.

While district comparison was performed with adjustments for the differences in sample composition, there might be confounders, such as psychosocial factors, that could not be adjusted for. Last but not least, the list of infrastructure, social amenities and services was not exhaustive, and some elements of service provision could only be shown in terms of quantity but not quality.

The use of the WHO age-friendly checklist was an arbitrary choice, to enable comparison between different populations. We did not develop an assessment tool de novo based on other country’s age-friendly neighbourhood experiences, although there are many recent examples. In Finland, sense of autonomy in outdoor activities and life-space mobility is important in maintaining mobility in old age.[28] Neighbourhood cohesion was a protective factor for risk of stroke death,[29] so that a detailed assessment of this characteristic may be relevant in the assessment of age-friendliness. Similarly neighbourhood social cohesion and social capital promote well-being of older adults in the community,[30] and relationships exist between frailty, neighbourhood security, social cohesion and sense of belonging.[31] The recent report from the age-friendly city movement in London emphasized provision of a minimum level of infrastructure, in addition to which there should be initiatives to enhance a sense of community, building intergenerational links, in particular to enhance the contribution of older people. In other words people of all ages should be engaged.[32] Other than a bottom up approach in engaging older people themselves, there is also a need for evaluation. Another shortcoming of the WHO checklist approach is that there may not be a ‘threshold’ for what is desirable, if subjective perceptions are influenced by factors such as socioeconomic status. It may be better to assess characteristics such as quality of life and social cohesion in addition. The results of this study suggest that this may be a better future approach in promoting the concept of age-friendliness.
